# Web-Based DICOM Viewers: A Survey and a Performance Classification

**DOI:** 10.1007/s10278-024-01216-5

**Published:** 2024-09-30

**Authors:** Hugo Pereira, Luis Romero, Pedro Miguel Faria

**Affiliations:** Instituto Politécnico de Viana do Castelo, Viana do Castelo, Portugal

**Keywords:** DICOM, Web-viewer, Med3WEb, OHIF, PostDICOM, 3D, 2D, MEDDream

## Abstract

The standard for managing image data in healthcare is the DICOM (Digital Imaging and Communications in Medicine) protocol. DICOM web-viewers provide flexible and accessible platforms for their users to view and analyze DICOM images remotely. This article presents a comprehensive evaluation of various web-based DICOM viewers, emphasizing their performance in different rendering scenarios, browsers, and operating systems. The study includes a total of 16 web-based viewers, of which 12 were surveyed, and 7 were compared performance-wise based on the availability of an online demo. The criteria for examination include accessibility features, such as available information or requirements for usage, interface features, such as loading capabilities or cloud storage, two-dimensional (2D) viewing features, such as the ability to perform measurements or alter the viewing window, and three-dimensional (3D) viewing features, such as volume rendering or secondary reconstruction. Only 4 of the viewers allow for the viewing of local DICOM files in 3D (other than MPR(Multiplanar reconstruction)). Premium software offers a large amount of features with overall good performance. One of the free alternatives demonstrated the best efficiency in both 2D and 3D rendering but faces challenges with missing 3D rendering features in its interface, which is still in development. Other free options exhibited slower performance, especially in 2D rendering but have more ready-to-use features on their web app. The evaluation also underscores the importance of browser choice, with some browsers performing much better than the competition, and highlights the significance of hardware when dealing with rendering tasks.

## Introduction

In the fast-evolving landscape of modern medicine, the efficient management and interpretation of medical images are paramount to patient care [1]. Digital Imaging and Communications in Medicine, commonly known as DICOM [2], has emerged as the universal language of medical imaging, revolutionizing the way healthcare professionals access and analyze diagnostic images. DICOM was developed to address the challenges of interoperability in medical imaging, ensuring that images and associated patient data can be seamlessly shared between various medical equipment, such as X-ray machines, MRI (Magnetic resonance imaging) scanners, CT (Computed tomography) scanners, and healthcare information systems. This standardized format allows healthcare professionals to access, view, and analyze medical images consistently, regardless of the manufacturer or origin of the equipment. DICOM files typically contain both the image data itself and important metadata, such as patient information, study details, and imaging parameters [2]. This rich set of information ensures that medical images are not only visually accessible but also correctly interpreted within the context of a patient’s medical history.

**Table 1:** DICOM standards evolution (1995–2024)

Year	Description
1995	Protocols for nuclear medicine, X-ray angiography, and ultrasound were introduced to DICOM to support cardiology imaging demands, and the DICOM Standards Committee was reorganized to represent all medical specialties that use imaging
1996	With the addition of the Modality Worklist service by DICOM, workflow management in the imaging department was standardized
1997	Radiation therapy information objects were added
1999	Endoscopy and dermatology were included in the standard, and it was made possible for image annotations to be presented consistently across display systems
2000	Further standardization on structured data, analytic results, and clinical observations, and addition of internet security mechanisms through secure communication profiles
2001	Mammography CAD (Computer Aided Detection) added, and new security improvements
2003	Multi-frame enhanced image formats added, with the objective to support future generations of MR and CT imaging techniques. Support for DVD (Digital Versatile Disc) media also added
2004	WADO (Web-access to DICOM objects) added, dentistry and ophthalmology joined DICOM, support for USB and flash memory media
2005	Radiation Dose Structured Reports (RDSR) added for x-ray-based imaging
2006	Deformable spatial registration added
2007	Radiation Dose for CT (RDSR) added
2008	Breast tomosynthesis (“3-D mammography”) added
2009	3D ultrasound added
2010	Whole slide imaging and surgical planning information objects added
2011	Bluray media exchange added
2013	Second-generation RESTful web services created to retrieve, store, and query DICOM images and rebranding of web services to DICOMweb™
2014	Radiopharmaceutical Radiation Dose Reporting (RRDSR) added
2015	Server-based Rendering added to WADO-RS, enabling web clients to make requests for the presentation of DICOM images and video. Tractography Results storage added. Imaging report templates using the HL7 Clinical Document Architecture (CDA) added. Wide-Field Ophthalmic Photography images, brachytherapy Delivery Instruction objects, and support for AVC/H.264 (MPEG-4) video added
2016	CT Protocol Storage, small Animal Acquisition Context headers, and HEVC/H.265 (MPEG4) video support added. Adult Echo Measurement SR objects updated/simplified
2017	Volumetric and Blending Presentation States, Patient Dose Estimate reports (P-RDSR) based on RDSR data for individual patients and Ophthalmic OCT angiography (OCT-A) imaging added. NCI AIM to DICOM SR transcoding is defined to facilitate the management of image markup
2018	3D Manufacturing - STL encapsulation added image-based medical 3D-printing workflows. Contrast Injection SR and Multi-Energy CT Image Storage added TLS ciphersuites updated to match security recommendations
2019	DICOMweb documentation re-organized. DICOMweb Thumbnails service and Realtime Video Streaming (DICOM-RTV) added
2020	Dermoscopy, Neurophysiology Waveform, and 2nd Gen. Other non-C-Arm RT supplements added to the DICOM Standard. Further support for Virtual Reality, Augmented Reality, and Mixed Reality was added
2021	Whole Slide Imaging Annotation, and cone-beam CT (CBCT) acquisition support added
2022	TLS security was updated, and the DICOM Conformance Statement was revised
2023	Support added for Confocal Microscopy, Photoacoustic Imaging, 32-bit ECG Waveform, and JPEG 2000 (HTJ2K)

DICOM viewers represent the bridge between cutting-edge medical imaging technology and the need for seamless data interoperability across various healthcare devices and systems [3]. These versatile software applications are designed to unlock the full potential of DICOM, allowing healthcare providers to access, manipulate, and analyze medical images with precision and ease. In doing so, they facilitate collaborative decision-making, accelerate diagnoses, and improve patient outcomes [2–4].

In addition to traditional DICOM viewers, there is a growing demand for DICOM web viewers. These web-based applications provide users with the flexibility to access and analyze medical images from any location with an Internet connection. DICOM web viewers have become especially valuable in the area of telemedicine and remote healthcare services [5]. They enable healthcare providers to securely access and share medical images with colleagues and specialists around the world in real-time. With the ability to view and manipulate DICOM images through a web browser, healthcare professionals can collaborate effectively, make faster diagnoses, and offer timely interventions, ultimately enhancing patient care.

This article will explore the most recent and popular DICOM viewers, including a literature review on some traditional desktop applications and a survey on the innovative DICOM web viewers. The article will analyze their capabilities, features, and performance, using DICOM images provided by the enterprise IDENTISOFT. This assessment will help users choose the best tools for their specific needs. In the “State of the Art” section, the article goes over the DICOM standard in detail, DICOM viewers, and surveys that have been done on them in the past. In the “Viewers and Technologies” section, the article presents the technologies used in this survey and the libraries that they use, in the “DICOM Security and Privacy” section, the article goes over the metrics used in the survey, in the “Metrics” section, the survey is presented, in the “DICOM Viewer Survey and Performance Comparison” section the performance comparison, followed by the “Results and Discussion” section discussing the results and the conclusion in the “Conclusion” section.

## State of the Art

The National Electrical Manufacturers Association (NEMA) established a joint committee in 1983 with the goal of creating a standard for connecting displays and related equipment to various manufacturers’ medical imaging equipment [2]. ACR/NEMA Standard Version 1.0, the initial version, was released in 1985. The original version 1.0 of the standard was followed by two updates, released in October 1986 (No. 1) and January 1988 (No. 2). Versions 1.0 and 2.0 both worked with point-to-point connections, which posed a challenge for communication networks that do not employ wholly dedicated channels. A revision of the standard ensued, leading to the emergence of DICOM Version 3.0 in 1993 [2, 6, 7]. This revised iteration revolutionized imaging system communications by transitioning from a point-to-point connection environment to a networked one. DICOM Version 3.0 facilitates cost-effective connectivity across expansive geographic areas, leveraging existing network infrastructure. In 2004 Picture Archiving and Communication System (PACS) originated for usage in healthcare facilities to manage and store the data generated within radiology departments [8]. PACS established a standardized method for obtaining, storing, and sending medical pictures and adopted the DICOM standard which specifies the handling, storing, and transmission of medical imaging data. The DICOM Standard has undergone numerous revisions since its 1993 publication and its history is publicly available [7] as shown in Table 1, from 1995 to 2024.

These updates/changes are done mainly through working groups. Some of the groups with importance to radiology include cardiac and vascular information and ultrasound [6]. When the changes are approved, they become an official part of the standard. Having gone through these changes has made DICOM the most widely implemented and supported communications standard for medical imaging [6].

Web-based DICOM viewers have been making substantial progress through the years as a result of rising demand, usage, and potential for browser-based software. Besides browser viewers, there is plenty of standalone software for viewing and processing DICOM-based images such as MITK (The Medical Imaging Interaction Toolkit), released in 2016 and is now on its newest version v.2023.04. It was created with the goal to drastically cut down on the time and effort needed to construct specifically tailored/custom, interactive applications for medical image analysis [9]. It uses two libraries ITK (Insight Toolkit) and VTK (The Visualization Toolkit). ITK is an open-source software toolkit developed by the US National Library of Medicine of the National Institutes of Health in the year of 1999 [10]. It offers a wide range of medical image processing techniques and analysis algorithms, but it does not include algorithms for data visualization. VTK is an open-source, freely available software system for 3D computer graphics, modeling, image processing, volume rendering, scientific visualization, and information visualization. These two are some of the most popular libraries which are often used together to create DICOM viewing software [11].

There have been many studies in the past that evaluate many of the older DICOM viewers, such as “Evaluation of commercial PC-based DICOM image viewer” which was published by Honea et al. in 1998 [12]. The study evaluated essential features like various windowing levels, measures of distance, and angles in the best free software that was available at the time. A study in 2003 by Horii made an analysis of viewing capabilities using supported DICOM object types, image processing techniques, and picture exporting capabilities [13]. Liao et al. carried out the first systematic evaluation in 2008 through which 21 software projects containing a GUI (Graphical User Interface) were examined in terms of “support, portability, workability, usability, data import, data export, header viewing, two-dimensional, and three-dimensional imagine viewing” [14]. Most categories were divided by their “yes/no” capabilities, with a few exceptions which were graded based on percentage value. The previously mentioned surveys are rather old and outdated, and have had newer surveys based on their work such as “A Survey of DICOM Viewer Software to Integrate Clinical Research and Medical Imaging” [15] in 2015 which performs a comprehensive evaluation of state-of-the-art DICOM viewer software for medical usage. They defined use cases for DICOM with a certain level of abstraction, these use cases being split into: Central viewing where data is gathered from PACS and two-dimensional (2D) functionality is what’s needed rather than sophisticated three-dimensional (3D) rendering; Decentral viewing where data is shared between sites [16] being a good fit for DICOM web viewers; Advanced viewing where in some circumstances, additional viewing features such as sophisticated 3D picture rendering and volumetric data processing is critical. Another survey in 2015 was made focusing on the loading of patient-specific 3D models [17], the survey uses a mix of qualitative methods such as subjective percentage values, and the Yes/No evaluation method. However, it’s important to note that even this survey is 6 years old, and new tech has been developed and released since its writing. The most recent research found is from 2020. The first is focused on free DICOM viewers for use in veterinary medicine, carried out by Andreas Brühschwein [18], and has a different approach to the previous ones, as a group of observers was selected and asked to perform some selected tasks with the various software and score the operability of each tool using a scale of 1 to 10. This study also found that gender, computer skills, veterinary degree, and experience did not affect the grading of the viewers significantly. The second study is an “Evaluation of free, open-source, web-based DICOM viewers for the Indian national telemedicine service” [19] and goes over 6 viewers with web capabilities and available online documentation and demo. It is the first study to mention an old version of OHIF although this version did not have any 3D capabilities.

## Viewers and Technologies

In this section, the article will provide a brief overview of the 16 web-viewers considered for the survey and what technologies/libraries they use. This compilation of prominent web-based DICOM viewers is the result of diligent research and evaluation. The selection approach involved thorough exploration/searches on popular search engines, GitHub, and a review of viewers used on previous surveys. Some of the chosen tools have standalone versions; however, this survey will only analyze their web-based parts. Any available standalone versions will be mentioned in the survey table. To be included in the survey, each viewer must pass a screening process. The viewer must either be free or, have a free trial available for usage, not be a duplicate entry, and have a working online demo, or be able to run on a local server.

### Open Health Imaging Foundation

- **Programming Language:** JavaScript
- **Description:** The Open Health Imaging Foundation, commonly referred to as OHIF, is a prominent organization dedicated to advancing healthcare through innovative medical imaging technologies [20], their viewer is a versatile and open-source medical imaging viewer designed to facilitate the visualization and analysis of medical images. They have recently released OHIF v3 which now uses the also newly released cornerstone3D which builds on top of cornerstonejs (a web-based medical imaging platform) to include 3D viewing capabilities.

### Med3Web

- **Programming Language:** JavaScript
- **Description:** Med3Web is a high-performance web application for sophisticated medical volumetric data display (in 2D and 3D modes) [21]. The most recent version is compatible with mobile browsers (Android Chrome) and desktop browsers that support WebGL. It uses DICOMParser from cornerstonejs, Daikon, a JavaScript DICOM reader, Xtk, a framework/toolkit for scientific and medical visualization that makes use of WebGL to provide sophisticated 3D medical visualization, and Three.js, a JavaScript framework for WebGL visualization.

### Papaya Online DICOM Viewer

- **Programming Language:** JavaScript
- **Description:** Papaya is a medical research picture viewer that runs only on JavaScript and Daikon for DICOM support [22]. Supported data types for this orthogonal viewer include overlays, atlases, DTI (Diffusion Tensor Imaging), and VTK(Visualization Toolkit) surface data. With numerous display, menu, and control options, the Papaya UI is highly adaptable. It can be used locally as a shared file or on a web server.

### DICOM Web Viewer

- **Programming Language:** JavaScript
- **Description:** The DWV (DICOM Web Viewer) is a free and open-source medical image viewer with a minimal footprint [23]. It solely employs the use of JavaScript and HTML5 allowing it to operate on multiple platforms

### Weasis

- **Programming Language:** Java for standalone, JavaScript for web
- **Description:** Weasis is a highly modular standalone and web-based DICOM viewer with a multifunctional architecture [24]. It is a widely used clinical viewer in healthcare, with hospitals, health networks, multi-center research trials, and patients as their targeted consumers. It is a cross-platform open-source software and through the OpenCV library, it enables high-quality renderings with high performance.

### IMAIOS DICOM Viewer

- **Programming Language:** Not publicly disclosed
- **Description:** IDV - IMAIOS DICOM Viewer is a medical image viewer app for web browsers and Android devices [25]. It accepts all DICOM files, such as ultrasound, scanner, MRI, PET, etc. The app includes numerous tools for manipulating and measuring graphics, such as scrolling and image alteration.

### PostDICOM

- **Programming Language:** Not publicly disclosed
- **Description:** PostDICOM Viewer is a medical imaging software application that allows the examination and handling of DICOM files [26]. It provides a user-friendly platform for healthcare practitioners and radiologists to access, evaluate, and analyze DICOM pictures, with capabilities such as multi-planar reconstruction, measuring tools, and the ability to annotate and store patient data. It makes medical picture interpretation easier, making it a crucial tool for medical diagnosis and patient care. It also provides its own PACS server in the form of cloud storage allowing for any photos obtained from patients to be directly uploaded to the server and then accessed from anywhere with the appropriate credentials.

### Voxelx

- **Programming Language:** Not publicly disclosed
- **Description:** VoxelX was a medical imaging platform and healthcare ecosystem that uses blockchain and artificial intelligence (AI) to enhance how doctors, researchers, and patients engage with medical data. This technology was intended to make medical images and related data more accessible and safe by streamlining storage, sharing, and analysis [27]. VoxelX seeked to improve medical imaging interpretation, diagnostic accuracy, and the interchange of medical information by combining 3D visualization, deep learning algorithms, and blockchain technology. The project however is no longer accessible and no new news has been given by the development team since 2018, leading to its exclusion from the survey.

### FViewer

- **Programming Language:** Not publicly disclosed
- **Description:** FViewer is a Free, online cloud file viewer that takes no download or installation and supports 12 file formats one of which is the DICOM format [28].

### Open DICOM Viewer

- **Programming Language:** Java
- **Description:** Open DICOM Viewer (ODV) is a lightweight multi-platform program for viewing DICOM images. It is portable as it may be used immediately from removable media, and supports web view display for displaying DICOM images on web pages using Java technologies [29]. This Viewer was built using Java. It was tried on two Windows 11 machines and results indicate that it no longer works on the newer Windows version. On Windows 10 the application opens but it was not capable of loading DICOM 3.0 files.

### Osimis

- **Programming Language:** JavaScript
- **Description:** The Osimis Basic Web Viewer extended Orthanc an open-source DICOM server with a Web viewer of medical images [30], with more advanced features. It has however been acquired by “Deepc” and is no longer available for free nor does the new version feature a free trial version available and was excluded from the survey.

### DICOMBinator

- **Programming Language:** JavaScript
- **Description:** DICOMBinator was created over 24 h for the SXSW Interactive Hackathon [31]. “Through an easy-to-use interface, this web-based tool enables users to examine and annotate DICOM medical pictures” The app was built using JavaScript allowing “instantaneous dissemination of comments and visual annotations to other users” enabling then instantaneous communication. This application however was built with node 0.6 and this version requires 32-bit, an attempt was made to run the web app using node 0.8.28 but without success, the app also has a demo on its GitHub page however it no longer works, leading to its exclusion from the survey.

### Hida DICOM Viewer

- **Programming Language:** JavaScript
- **Description:** Hida is a JavaScript DICOM viewer and analysis tool. It was developed as part of a research internship at the Academic Medical Center in Amsterdam [32]. Its goal was to provide a future-proof rebuild of a Liver Uptake measurement algorithm [32] but grew as a general-purpose web-based DICOM tool. No demo was provided and the viewer failed to install on two different computers, leading to its exclusion from the survey.

### Slice:Drop Online DICOM Viewer

- **Programming Language:** JavaScript
- **Description:** Slice:Drop (Sdrop) is a web-based application that allows for the instant viewing of scientific and medical imaging data in 3D [33]. It supports a variety of file formats including DICOM. It was programmed with JavaScript using WebGL for the rendering.

### Oviyam

- **Programming Language:** Java
- **Description:** Oviyam is a web-based open-source DICOM Viewer [34], it is pre-packaged for deployment with JBoss. It was built using the dcm4che toolkit and script.aculo.us framework and programmed in the Java Language.

### DicomLibrary and MedDream

- **Programming Language:** JavaScript
- **Description:** DicomLibrary is a free online medical DICOM image or video file-sharing service for educational and scientific purposes [35]. All DICOM files are anonymized before they are uploaded into the cloud, no personal information is ever revealed ensuring the user’s or patient’s privacy. It uses the MedDream DICOM viewer, an “HTML 5 zero-footprint DICOM VIEWER” aimed at making diagnoses, viewing, archiving, and transmitting medical images [35, 36]. Together these tools can be used to upload and view your own DICOM files.

## DICOM Security and Privacy

In this section, the article will provide a brief overview of how DICOM, and each viewer, handle the security and privacy of the files.

An important question when using DICOM is how the standard holds itself in the modern world of IT, with data in the clouds, hackers accessing our systems, and ransomware in hospitals these files must be secure as they contain personal private information [7]. Regarding security and privacy, DICOM is up to the task; however, real security and privacy hinge fully on how the standard is implemented, in both the products and the field deployment of these products.

Most DICOM items include images with related patient demographic and medical data that must be kept private [7]. One method to protect the privacy of these data is encryption. Although the encryption is not specifically specified by DICOM, a number of DICOM Standard features can help with encryption, such as the ability to read and transport encrypted DICOM objects to and from the recipient.

- On email DICOM defines how to encrypt the files using CMS encryption methods.
- On a traditional DICOM transfer mechanism (the DIMSE protocol), DICOM defines how to use an encrypted TLS connection.
- On DICOM transfer mechanism (DICOM web services), DICOM defines how to use an encrypted HTTPS connection.

It is crucial to remember that DICOM does not require encryption; rather, it only facilitates its usage. Health facilities have the option to build up a VPN-secured network and use unencrypted DICOM if the product vendors have chosen not to provide encryption, or even if they do [7].

Assessing the security of DICOM files on different online viewers entails looking at data encryption, access control, adherence to healthcare laws such as HIPAA(Health Insurance Portability and Accountability Act), and the hosting environment’s security measures.

Some of the proposed viewers have an online platform ready for usage with security measures implemented while others would need security measures implemented on the server side to ensure the privacy and safety of the files being used.

OHIF, MED3WEB, DWV, IMAIOS, PostDICOM, FViewer, SliceDrop, and MEDDream all provide HTTPS encryption on their online ready-to-use products. Except for the PostDICOM and MEDDream viewers, which allow the user to use cloud services, all of the DICOM files sent to the viewers are handled client-side only, ensuring the files’ privacy.

Most of these viewers however do not mention HIPAA compliance, out of them only PostDICOM and MEDDream claim full HIPAA compliance.

For the other viewers as there is no online demo, encryption and other security measures need to be implemented at the server level. HIPAA compliance will also rely on server-level implementation.

## Metrics

This section will go over the metrics used for the evaluation and comparison of the DICOM viewing software. The choice of metrics for evaluating DICOM viewing software is crucial in assessing the accessibility, functionality, and usability of such tools. The acquisition of these metrics involved a thorough review of scientific articles, surveys conducted by other researchers, and extensive online searches [13–15, 17–19]. The selection covers a broad range of aspects, ensuring an extensive evaluation. A total of 29 metrics were chosen to provide a comprehensive survey of DICOM viewing software. These metrics are separated into 4 categories, each with multiple sub-categories. The first category, accessibility, covers aspects such as registration, platforms, documentation, contact information, and whether the viewer is free. The second category, interface, assesses the available load options, cloud storage features, and whether the viewer is extendable. The third and fourth categories focus on 2D and 3D viewing, respectively, with subcategories covering their respective capabilities.

### Accessibility

Nine accessibility metrics were set that define how readily available the tool is and if useful information for the software is made available.

- **M1 - Registration Required** If the software/tool requires the user to go through a registration and login process to begin utilization.
- **M2 - Free / open-source** If the software is available to be used for free by any user, software with free trials will be considered paid.
- **M3 - Standalone Version** Some of the surveyed applications contain both a web version and a version that runs standalone on the user’s computer.
- **M4 - Multi-platform** Certain programming languages, like Java, are platform-agnostic. Platform-independent software can run on standalone systems, using Java runtime environments (JRE), or be delivered to client systems via Java Web Start.
- **M5 - Mobile** If the DICOM viewer offers a suitable graphical user interface (GUI), medical images can be viewed on mobile devices such as smartphones or tablets.
- **M6 - Documentation** Comprehensive written documentation for the software, such as manuals, is accessible.
- **M7 - Mail** A mailing list is provided to offer support through email correspondence.
- **M8 - Forum** A web-based forum is available to provide support.
- **M9 - Wiki** A user-accessible wiki webpage is provided for reference and assistance.

### Interfaces

Four interface metrics were set, used to facilitate communication with other systems required for the loading of DICOM files.

- **M10 - Load from local files/folders** Allow the loading of content directly from the machine via. DICOM files are stored in the computer or zip files containing them.
- **M11 - Load from URL** Web protocols such as web access to DICOM objects (WADO) service allow a system to provide access to DICOM objects to other systems directly from the web.
- **M12 - Cloud-Based Storage** The DICOM files/projects are stored in a cloud web server for usage at later dates without the need to have the original DICOM files on the computer.
- **M13 - Extendable** Extensibility refers to the software’s capability to be expanded and integrated with other systems to meet needs in medical imaging. Software developed with extensibility in mind allows developers to add new functionalities, modify existing ones, and seamlessly integrate with other healthcare applications.

### 2D Viewing

Eight 2D viewing metrics were set, they go over the viewing functionality and supported features of the various viewers.

- **M14 - Scrolling** Mouse scroll allows users to move to the next or previous image, zoom in and out, or swap view mode by simply scrolling with the mouse wheel.
- **M15 - Metadata** The header viewing functionality involves processing and showing the metadata of DICOM objects.
- **M16 - Overlay Information** Relevant data should be rendered as an overlay in the display window.
- **M17 - Windowing** The brightness and contrast of the displayed image are controlled via windowing.
- **M18 - Pseudo Colors** Pseudo-color look-up tables (LUT) convert image gray values to pseudo-colors.
- **M19 - Measurements** Measurements allow the drawing of content (e.g., lines) as well as the evaluation of geometric figures in the image such as distances or angles.
- **M20 - Annotations** Image viewing results (e.g., measurements, text annotations) should be saved for subsequent use.
- **M21 - Histogram** A graphical representation of the distribution of pixel intensity values within the medical image.

### 3D Viewing

Eight 3D viewing metrics were set, they go over the 3D viewing capabilities of the viewers.

- **M22 - MPR (Multi-Planar Reconstruction)** Multi-Planar Reconstruction refers to the process of reconstructing two-dimensional images in multiple planes from a three-dimensional dataset.
- **M23 - Crosshairs** Mouse interaction with the MPR viewports allows for on-click selection of the slice the user wants to see.
- **M24 - Segmentation** Tools for creating, editing, and visualizing segmentations, dividing a dataset into distinct regions or structures.
- **M25 - Secondary Reconstruction** Medical volume data is often collected along a single body axis.
- **M26 - Scroll Through** Moving inside through the 3D render can sometimes provide a better view of a particular part of the file.
- **M27 - Volume Rendering** Volume rendering is a visualization technique used in medical imaging to create 3D images from a series of 2D medical images.
- **M28 - Transfer Function** Transfer functionality can be used to map image voxel grey values to tissue opacity values.
- **M29 - Surface Generation** The creation of three-dimensional (3D) surfaces or models from medical imaging data. Allowing the user to visualize organs, bones, or other structures in a more lifelike and spatially accurate manner.

## DICOM Viewer Survey and Performance Comparison

The survey ascertains whether each DICOM Viewer tool supports or does not support each of the pre-established metrics. A positive sign (+) will denote affirmative support, while a negative sign (-) will indicate a lack of support. To establish the presence or absence of each feature within the applications they were tested, their documentation was meticulously reviewed, and the provided sample projects were examined. Should any of these methods confirm the existence of the feature, it is noted as a positive (+) in the survey. If all three methods fail to substantiate the feature’s presence, it is designated negative (-). The survey is displayed in Table 2.

**Table 2:** Web DICOM viewer survey

		OHIF	MED3WEB	Papaya	DWV	Weasis	IMAIOS	PostDICOM	FViewer	ODV	Sdrop	Oviyam	MedDream
Accessibility	No Registration	+	+	+	+	+	+	−	+	+	+	−	+
	Free / open-source	+	+	+	+	+	+^*8^	−	+^*8^	+	+	+	+^*8^
	Standalone Version	+	+	+	−	+	−	−	−	−	−	−	−
	Multi-Platform	+	+	+	+	+	+	+	+	−	+	+	+
	Mobile	+^*1^	+^*3^	−	+	−	+	+	+	−	+	+^*1^	+
	Documentation	+	+	+	+	+	−	−	−	−	−	−	+
	Mail	+	−	−	+	+	+	+	−	−	−	+	+
	Forums	+	+	+	+	−	−	−	−	+	+	+	−
	Wiki	−	−	−	+	−	−	−	−	−	−	−	−
Interface	Load from File	+	+	+	+	+	+	+	+	+^*4^	+	+	+
	Load from URL	+	+	+	−	+	+	+	+	+^*4^	−	+	−
	Cloud-Based Storage	+^*2^	−	−	−	−	−	+	−	−	−	−	+
	Extendable	+	−	+	+	+	−	+	−	−	−	+	+
2D Viewing	Scrolling	+	+	+	−	+	+	+	−	+	+	+	+
	Metadata	+	+	−	−	+	−	+	+	−	−	+	+
	Overlay	+	+	+	+	+	+	+	+	−	−	+	+
	Windowing	+	+	+^*5^	+	+	+	+	+	+	+	+	+
	Pseudo Colors	−	−	+	−	+	−	+	+	−	+	−	+
	Measurements	+	+	+	+	+	+	+	+	−	−	+	+
	Annotations	+	+	+	+	+	−	+	+	−	−	+	+
	Histogram	+	+	−	−	−	−	−	+	−	−	−	+
3D Viewing	MPR	+	+	+	−	+	+^*6^	+	−	−	+	−	+
	Crosshairs	+	+	+	−	+	−	+	−	−	−	−	+
	Segmentation	+	+	−	−	+	−	+	−	−	+	−	+
	Secondary Reconstruction	+^*7^	−	−	−	−	−	+	−	−	−	−	−
	Scroll Through	−	+	−	−	−	−	−	−	−	+	−	−
	Volume Rendering	+^*7^	+	−	−	−	−	+	−	−	+	−	+
	Transfer Function	+^*7^	+	−	−	−	−	+	−	−	+	−	+
	Surface Generation	+^*7^	+	−	−	−	−	−	−	−	+	−	+

*1 Works but not officially supported;

*2 Requires approval for cloud storage;

*3 Mobile has limited functionality;

*4 No longer works on modern machines;

*5 Sample app has limited filters;

*6 Mode available but failed local DICOM loading;

*7 Not available for local DICOM;

*8 Free but not open-source

Table 2 indicates that most viewers while relatively feature complete in the 2D viewing area, lack 3D viewing capabilities for DICOM files, with most only supporting MPR with Crosshairs. Slice Drop is an exception, meeting most of the 3D viewing metrics, but lacking most of the 2D ones. OHIF was the highest-scoring viewer in the survey. However, it’s important to note that its 3D viewing features are only accessible with example cases uploaded to the cloud by the developers, not locally loaded DICOM files. Taking the previous into account, OHIF met 26 out of the 29 defined metrics, MED3WEB and MedDream came second at 23, and PostDICOM third at 20, failing most of the accessibility metrics. Following these, Weasis and Papaya meet 19 and 18 metrics respectively, followed by SliceDrop with 15. Oviyam and DWV each meet 14, Fviewer meets 13 and finally, IMAIOS and ODV meet 12 and 7 metrics respectively. If 3D viewing is not included, the top performers are OHIF meeting 19 out of 21 metrics, followed by MedDream and Weasis, both with 17.

### Measuring Method

**Fig. 1:**
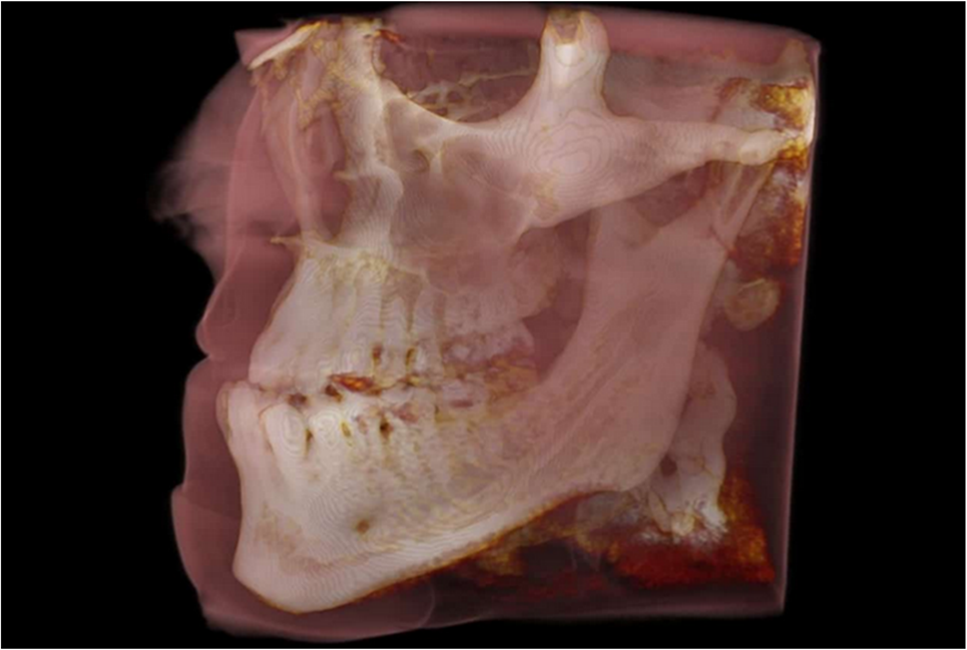
Visualization of the Class 3 malocclusion DICOM dataset

The forthcoming performance comparison uses a free and public data set featuring 517 DICOM images, accessible on the website Medimodel [37], showcasing a human skull afflicted with class 3 malocclusion. This dataset can be seen in Fig. 1 which depicts a 3D visualization of the DICOM files in the dataset.

**Table 3:** PostDicom test results

	Computer				
	A	B	C	D	E
**3D Rendering Test 1**					
Firefox	05.71s	06.54s	07.05s	−	−
Edge	06.82s	07.32s	09.03s	−	−
Chrome	05.89s	05.90s	07.34s	05.61s	06.60s
Safari	−	−	−	Fail	08.88s
**3D Rendering Test 2**					
Firefox	05.51s	06.31s	07.49s	−	−
Edge	06.86s	05.56s	06.12s	−	−
Chrome	05.70s	06.40s	04.23s	05.52s	06.16s
Safari	−	−	−	Fail	06.61s
**2D Rendering Test 1**					
Firefox	06.28s	06.52s	01.99s	−	−
Edge	04.15s	08.12s	02.61s	−	−
Chrome	03.05s	03.08s	15.87s	02.86s	04.21s
Safari	−	−	−	Fail	05.10s
**2D Rendering Test 2**					
Firefox	01.04s	03.27s	00.98s	−	−
Edge	04.11s	07.33s	02.15s	−	−
Chrome	00.87s	01.86s	15.96s	01.07s	01.66s
Safari	−	−	−	Fail	01.99s

The assessments will commence by loading the images into the designated software, followed by the initiation of the 3D rendering process. These tests will be performed on five distinct computers, which will be named, computers A, B, C, D, and E. Their specifications can be found in the Appendix file A, with computers A and B being modern machines with Windows 11, C an older computer with Windows 10, and D, and E being Mac computers.

The tests were conducted on 4 different browsers including Google Chrome, Microsoft’s Chromium-based browser known as ‘Edge,’ Firefox, and Safari. All tests were run on the computers with any other nonessential applications closed to optimize and streamline performance, allowing for reliable comparisons. Tests were only conducted on viewers with an online example ready for usage; viewers with only locally-run servers available were not considered for the performance comparison. The viewers OHIF, Med3EWeb, DWV, IMAIOS, PostDICOM, Fviewer, Slice Drop, and the DICOMLibrary+MedDream combo qualified for the performance comparison.

To gauge the duration of the software’s task completion, the computer screen was recorded using OBS (Open Broadcaster Software). The time intervals between the initiation of the loading process (once the files are selected, and the uploading process begins), and the display of the 2D image on the screen was measured. Additionally, the time intervals between pressing the 3D rendering button and the display of the render on the screen were recorded. Each table in this section features browsers in the first column, followed by a column for each computer. If a test is not conducted on a specific browser and computer, it is marked with (-); if a test fails to load the images, it is marked as (Fail), otherwise, the time it took to finish is displayed in seconds.

At least two tests were conducted per viewer, for both 2D and 3D (when available). The first test was conducted on a computer with its cache cleared or with the browser in private mode. The second test involves accessing the website for at least the second time, allowing the usage of the browser’s cache.

The tests should consider an error margin of around 300 milliseconds for the human reaction time [38] as the recordings still need to be paused for the time to be taken. The fastest 2D and 3D (when available) test times from tests 1 and 2 are underlined on each table, with a margin of error of 300ms from the quickest result.

### PostDICOM Testing

PostDICOM requires the user to first upload their DICOM files to their cloud, which, depending on the user’s upload speed, can take hours to upload larger datasets. The time taken to upload the files was not counted toward loading times or display times but was taken into account when the comparison with other viewers was made.

Following the successful loading of the 2D render, the time between the click of the 3D render button and the display of the 3D render was measured using the assistance of recording software. The time intervals can be seen in Table 3 in the 3D Rendering tests.

For the 2D Rendering times, the duration between the click of the upload file button and the conclusion of the loading process was taken using the assistance of recording software. The time for each test taken can be seen in Table 3 in the 2D rendering tests.

An extra test was done to confirm the abnormal 2D rendering results in Test 1 on (Firefox - C) and (Edge - A). Using the browsers in private mode on the C computer and using browser cache for computer A. The results were 04.53s C on Firefox and 04.08s A on Edge.

The extra test confirmed that although the faster loading experienced on computer C with Firefox was not as quick as initially observed, it was still faster than the loading speeds on other computers. Additionally, despite having better hardware, computer A performed worse than computer C on Edge and Firefox in the 2D tests.

Overall, based on the findings in Table 3, it can be concluded that Chrome is the best-performing browser for the stronger computers on postDICOM, exhibiting similar results to Firefox on 3D viewing and the best results in 2D viewing. For less powerful computers, Edge and Firefox emerge as better alternatives. Mac systems should also consider using Chrome when using postDICOM.

### OHIF Testing

OHIF does not currently allow for the 3D rendering of files locally. The page needs to be refreshed to show the 3D rendering, causing the web app to lose access to data. It’s not possible to download their available studies stored on the cloud and load them on other DICOM viewers. As a solution, to still allow comparison with the other viewers, a study that uses 642 instances designated “CT CHEST WITH IV COUNT” was picked as a replacement. This study will be loaded and rendered in 2D and, afterward, the hanging protocol to show the 3D Render ( &hangingprotocolId=mprAnd3DVolumeViewport) will be added. The page will be reloaded, and the time interval will be measured between the reload and the finish of the 3D render. It is important to note that OHIF performed the 2D and 3D rendering processes simultaneously as there is no way to separate the two currently.

**Fig. 2:**
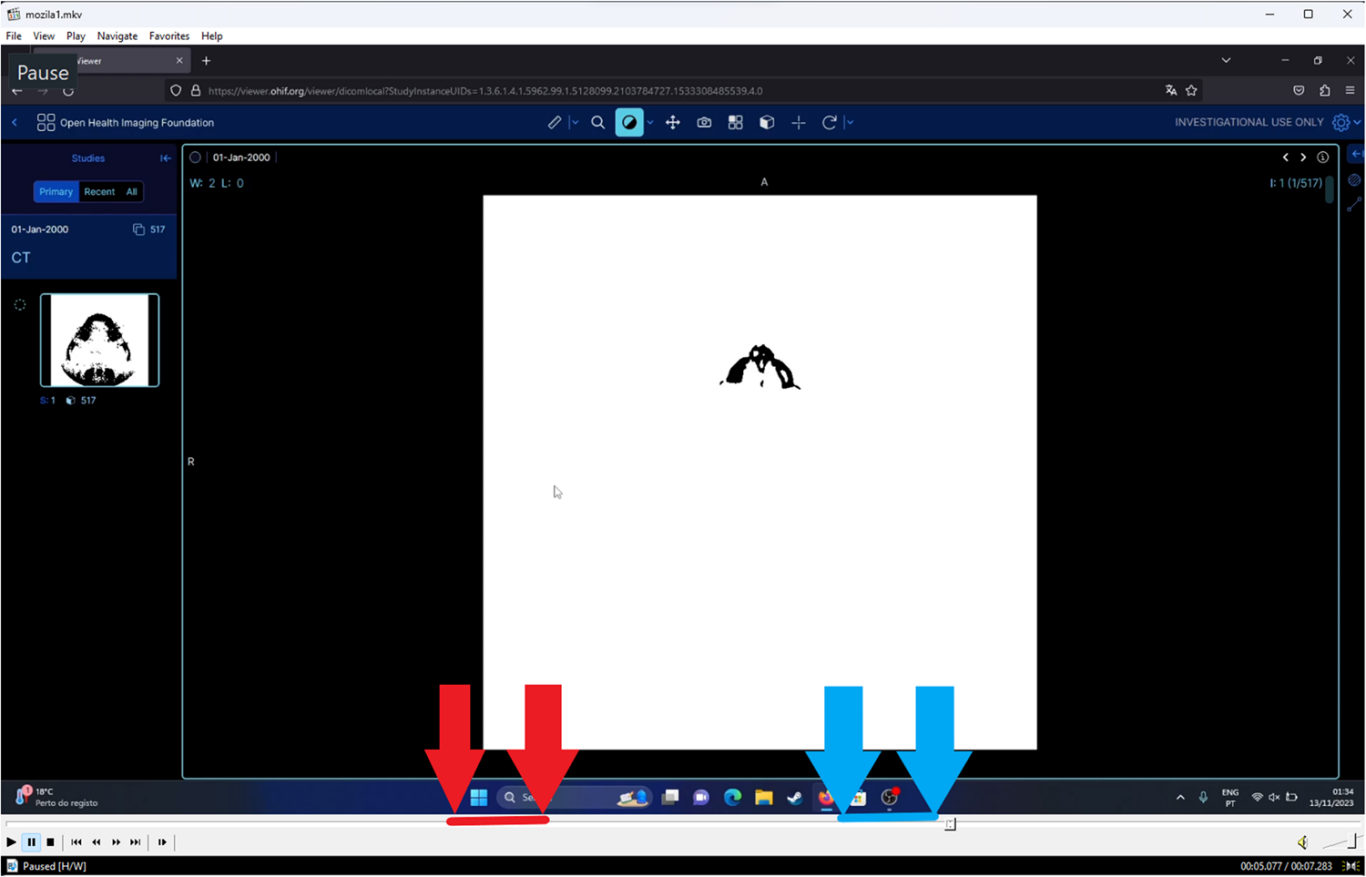
OHIF testing and measuring on the Firefox browser

Since OHIF loads the files, and then, gives the option to start the viewer, the 2D rendering tests were separated into two times, the time to load the files into the software, and the time to display the files on the screen, where [Result = (Loading time + Display time)]. The loading time was recorded between the click of the upload files button and the start of the viewer, while the display time was recorded between the click of the view button and the display of the 2D render. The time intervals for the first test are in the Appendix in B.2 displayed in Table 10.

The time acquisition process can be seen in Fig. 2 where the loading time is highlighted by the red arrows and the display time is highlighted by the blue arrows.

The time intervals for the second test are in the Appendix on B.2 displayed in Table 11. The full table with all the results can be seen in Table 4.

**Table 4:** OHIF rendering test results

	Computer				
	A	B	C	D	E
**3D Rendering Test 1**					
Firefox	02.02s	02.97s	07.05s	−	−
Edge	02.52s	05.04s	09.03s	−	−
Chrome	01.11s	01.51s	03.70s	02.70s	07.34s
Safari	−	−	−	Fail	Fail
**3D Rendering Test 2**					
Firefox	01.88s	02.34s	07.49s	−	−
Edge	03.07s	03.57s	06.12s	−	−
Chrome	00.93s	00.97s	03.16s	02.69s	04.23s
Safari	−	−	−	Fail	Fail
**2D Rendering Test 1**					
Firefox	01.18s	00.93s	01.99s	−	−
Edge	01.43s	01.47s	02.61s	−	−
Chrome	00.90s	01.24s	02.56s	02.27s	02.61s
Safari	−	−	−	Fail	Fail
**2D Rendering Test 2**					
Firefox	00.64s	00.92s	00.98s	−	−
Edge	01.47s	01.17s	02.15s	−	−
Chrome	00.72s	00.64s	01.29s	01.31s	01.62s
Safari	−	−	−	Fail	Fail

Overall, based on the findings in Table 4, Chrome and Firefox perform similarly in the 2D viewing area, with Edge lagging behind the alternatives and Safari not supported by the viewer. When looking at 3D viewing Chrome performed better in all parameters regardless of machine making it the go-to browser for this platform. Based on Tables 10 and 11 in annex A, it can be concluded that both the loading time and the display time benefit from the browser cache.

### IMAIOS Testing

IMAIOS tests begin with clicking the upload file button and end with the completion of the loading of the last file. The tests can be seen in Table 5.

Based on the data in Table 5, Chrome emerges as the better performer overall, except for Computer C, where Firefox was the faster loader. Chrome should be considered for stronger computers and Firefox for older, or weaker, computers. Additionally, Edge’s results were exceedingly slow for any computer other than A.

### DWV Testing

The DWV tests begin with clicking the upload file button and end when all the files have finished loading (indicated by the completion of the loading bar).

**Table 5:** IMAIOS rendering test results

	Computer				
	A	B	C	D	E
**2D Rendering Test 1**					
Firefox	03.23s	13.59s	20.09s	−	−
Edge	03.54s	246.84s	276.01s	−	−
Chrome	01.82s	12.83s	29.69s	22.37s	28.99s
Safari	−	−	−	46.42s	38.58s
**2D Rendering Test 2**					
Firefox	03.44s	13.61s	22.04s	−	−
Edge	07.79s	241.89s	437.11s	−	−
Chrome	01.79s	16.06s	29.88s	21.31s	28.96s
Safari	−	−	−	37.50s	36.74s

**Table 6:** DWV rendering test results

	Computer				
	A	B	C	D	E
**2D Rendering Test 1**					
Firefox	04.46s	01.30s	12.14s	−	−
Edge	06.57s	08.37s	21.89s	−	−
Chrome	03.67s	01.02s	12.59s	16.02s	15.86s
Safari	−	−	−	Fail	15.47s
**2D Rendering Test 2**					
Firefox	03.97s	01.34s	13.03s	−	−
Edge	06.68s	08.40s	22.16s	−	−
Chrome	01.03s	01.18s	12.07s	15.43s	15.74s
Safari	−	−	−	Fail	15.04s

In Table 6, Chrome emerges as the faster option overall, but it lags slightly behind Firefox on computer C. Additionally, Edge’s performance was slower compared to the alternatives.

### FViewer Testing

The FViewer tests begin with clicking the upload file button and end when all the files have finished loading (indicated by the processing wheel stopping).

Additional tests were conducted on computer A on Edge to confirm faster load times compared to the other computers, and on computer C, also on Edge, to confirm slower loading times experienced on this browser compared to the others. The caches were not cleared, and the results were as follows: 27.65s for computer A and 53.41s for computer C.

Based on the data in Table 7, it is evident that FViewer does not benefit from browser cache usage, as the second results were consistently slower, or, similar across all browsers except Safari. Notably, Safari performed slightly better than Chrome on MAC computers. Firefox failed to function with FViewer on all tested computers. Additionally, Edge’s performance was notably slower compared to the alternatives.

### Med3Web

Because MED3WEB loads the files before giving the option to choose between 8-bit and 16-bit rendering, the 2D rendering tests were divided into two time intervals: the time to finish file processing (Loading time), and the time from the selection of the processing method to the display of the rendering (Display time). Similar to the procedure used with OHIF, the overall result was calculated as [Result = (Loading time + Display time)]. For consistency, the 16-bit rendering method was selected across all devices and browsers. The time intervals for the first test are provided in the Appendix on B.2, displayed in Table 12. The time intervals for the second test are in the Appendix on B.2, displayed in Table 13.

After the successful loading of the 2D render, the time between clicking the 3D render button and the display of the 3D render was measured using recording software. The results can be seen in Table 8 on 3D rendering Test 1 and 2.

Accidental inputs were made during the testing of the 3D rendering on device A, specifically while using Edge by clicking the 3D rendering UI. To ensure a fair comparison with other devices, an additional test was conducted on Edge without clearing the browser cache. The result was as follows: 34.21s A Edge;

The full table with all Med3Web’s results can be seen in Table 8.

**Table 7:** FViewer rendering test results

	Computer				
	A	B	C	D	E
**2D Rendering Test 1**					
Firefox	Fail	Fail	Fail	−	−
Edge	12.13s	32.09s	26.77s	−	−
Chrome	04.63s	06.26s	08.83s	09.13s	12.91s
Safari	−	−	−	10.08s	13.90s
**2D Rendering Test 2**					
Firefox	Fail	Fail	Fail	−	−
Edge	31.37s	31.06s	54.61s	−	−
Chrome	05.22s	06.47s	10.63s	08.75s	13.61s
Safari	−	−	−	08.46s	12.05s

**Table 8:** Med3Web test results

	Computer				
	A	B	C	D	E
**3D Rendering Test 1**					
Firefox	04.96s	05.56s	17.08s	−	−
Edge	49.21s	36.93s	98.49s	−	−
Chrome	02.73s	03.63s	18.81s	04.58s	05.12s
Safari	−	−	−	Fail	05.52s
**3D Rendering Test 2**					
Firefox	03.13s	05.29s	19.48s	−	−
Edge	55.78s	36.76s	96.06s	−	−
Chrome	02.54s	03.13s	20.19s	04.25s	04.84s
Safari	−	−	−	Fail	04.51s
**2D Rendering Test 1**					
Firefox	09.23s	09.89s	28.27s	−	−
Edge	152.19s	155.19s	309.14s	−	−
Chrome	07.42s	08.22s	21.40s	15.56s	15.87s
Safari	−	−	−	Fail	14.04s
**2D Rendering Test 2**					
Firefox	10.53s	07.42s	23.82s	−	−
Edge	151.97s	156.02s	302.62s	−	−
Chrome	07.40s	14.99s	21.20s	15.29s	15.96s
Safari	−	−	−	Fail	12.85s

Table 8 indicates that Chrome performed the best in both 2D and 3D rendering on Windows machines, although it lagged behind the Safari browser on computer E. Additionally, Edge’s rendering times were significantly longer on all three Windows computers, making it nearly unusable.

Based on Tables 12 and 13 in Appendix B.2, it can be concluded that the time needed to load the images is consistently longer than the time to display the images on the screen and that no improvements are found with browser cache.

### MedDream Testing

If the user intends to use MedDream with their own study, the viewer requires users first to upload their DICOM files to the DICOMLibrary cloud. Depending on the user’s upload speed, the uploading process can take multiple hours for large datasets. The time taken to upload the files was not counted toward loading or display times but was considered during comparisons with other viewers.

After successfully uploading the DICOM dataset to the web, DICOMLibrary generates links to access and view the files on MedDream. The time interval between opening the link and displaying the files in 2D was measured and is displayed in Table 9 in the 2D tests.

Once the viewer finishes the loading process, the time between clicking the “MPR - Mist Oblique” button and displaying the 3D render of the dataset was measured and is displayed in Table 9 in the 3D tests.

A third test was conducted to confirm the improved loading times during the second test on the browsers on Computer E. The results showed 10.87 s on Chrome and 12.81 s on Safari, confirming the improved results with cached data.

**Table 9:** MedDream test results

	Computer				
	A	B	C	D	E
**3D Rendering Test 1**					
Firefox	14.35s	16.09s	46.10s	−	−
Edge	06.35s	34.16s	Fail	−	−
Chrome	06.34s	07.12s	Fail	−	Fail
Safari	−	−	−	−	Fail
**3D Rendering Test 2**					
Firefox	14.43s	16.33s	44.32s	−	−
Edge	06.59s	29.89s	Fail	−	−
Chrome	06.01s	07.17s	Fail	−	Fail
Safari	−	−	−	−	Fail
**2D Rendering Test 1**					
Firefox	02.16s	02.28s	03.18	−	−
Edge	01.58s	02.70s	03.35s	−	−
Chrome	02.37s	02.69s	03.20s	-	36.60s
Safari	−	−	−	−	51.50s
**2D Rendering Test 2**					
Firefox	01.71s	01.37s	02.67s	−	−
Edge	01.19s	01.76s	02.59s	−	−
Chrome	01.03s	01.62s	02.22s	−	08.64s
Safari	−	−	−	−	15.01s

Table 9 indicates that Chrome performed the best in both 2D and 3D rendering tests. However, it failed to load the 3D render of the file on computer C. Only the Firefox browser was able to obtain a 3D render on computer C. The 3D rendering tests ran for fifteen minutes on computer C before being marked as failed. Notably, the loading bar reached halfway around the one-minute mark and did not progress further on both browsers.

### Drop Slice Testing

**Fig. 3:**
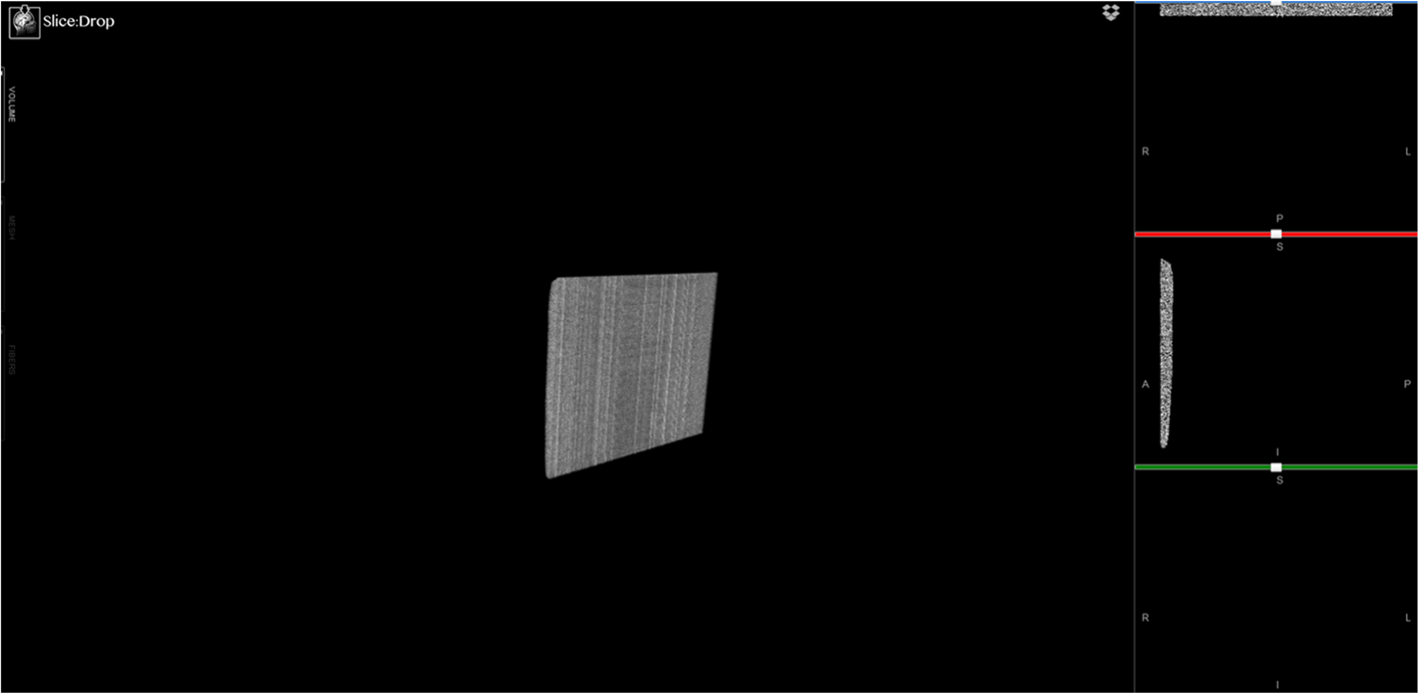
3D Visualization of the Class3Malocclusion Dataset on Slice Drop

Slice Drop failed to properly display the dataset on all computers and browsers in both 2D and 3D, instead displaying the result depicted in Fig. 3. Although a smaller test using a twenty-file dataset led to the display working correctly, the discrepancy in size was too large, resulting in the testing being marked as unsuccessful.

## Results and Discussion

The performance tests on various DICOM viewers yielded insightful results across different rendering scenarios, browsers, and operating systems. PostDICOM saw the best results across all viewers for local 3D rendering on computer C proving an excellent choice for weaker computers. On this viewer, Firefox and Chrome exhibited similar performance, while Edge lagged slightly behind. In the area of 2D rendering in PostDICOM, a substantial improvement was observed during the second rendering, particularly noteworthy on Firefox for Computer C. Interestingly, Chrome outperformed Firefox on more powerful machines (A and B) but struggled on the older machine (C) in the 2D rendering tests. Additionally, newer versions of Safari are required to use this browser on PostDICOM specifically versions above 15.1.

Regarding the OHIF viewer, the 3D rendering interface, although not integrated into the user interface, can be accessed by adding a “hanging protocol” to the project link. However, issues surfaced when using locally loading DICOM files, disrupting rendering upon the reloading of the page. Developers have communicated future implementation, underlining that “An interface for 3D rendering” is planned for the future. The 3D rendering tests were conducted using a different study with 642 instances instead. The results showed that, even with a larger study, OHIF remains the fastest available web browser for 3D rendering. While no significant differences were noted between Firefox and Chrome, Edge consistently lagged slightly behind on all computers. Additionally, Safari for iOS systems does not work with the current version of OHIF, regardless of the Safari version. Display times for OHIF proved consistently superior to all other viewers, especially considering that files do not need to be uploaded to the OHIF cloud before usage.

In the case of the DWV Viewer compatibility issues arose, with DWV failing on Safari 13.1.2. Additionally, excluding the PNG file present in the dataset was necessary for proper folder loading on Devices A and B. This issue did not occur on Computer C, suggesting a connection to Windows 11. The viewer lags behind and offers less functionality than more modern counterparts such as OHIF.

IMAIOS Viewer showed large discrepancies during the loading of DICOM files. Notably, Edge exhibited better performance on computer A when compared with all other machines which had very long load times. However, Edge still fell behind Chrome and Firefox. A combination of a desktop computer with Windows 11 and very good hardware proves essential for getting good results from this viewer. It’s important to note that even the best results in IMAIOS lag behind OHIF.

FViewer performed worse than IMAIOS and DWV on Windows but better than these viewers on iOS. However, it still performed worse than OHIF and PostDICOM across all parameters. Additionally, this viewer separates all images into individual tabs, making it less convenient for users to scroll through.

Med3Web’s 3D rendering on computer A initially displayed slower results due to an accidental input. However, without this input on test 3, the results mirrored those on computer B. Older versions of Safari (13.1.2 or below) do not work with the software, and Edge had poor results. Chrome and Firefox, on the other hand, performed similarly with the content loading reasonably fast, however, even on stronger machines, a one to two-second delay was observed when altering or moving the render on the 3D side of the app. The 3D rendering on Med3Web proved slower than that provided by PostDICOM and OHIF. However, it’s important to note that Med3Web is free and already offers the interface for volume rendering, unlike OHIF. In contrast to alternative viewers, Med3Web allows users to selectively determine the quality of the rendering process and opt for accelerated 3D rendering, having more customization options.

The DICOMLibrary and MEDDream combo proves to be a good free competitor to PostDICOM, providing most of the same features, including cloud storage, with better overall performance in 2D and slightly worse in 3D. However, on the Windows 10 computer, the viewer failed to load the 3D render of the dataset on Edge and Chrome. The loading bar would finish, but the display would not render. The test ran for over 30 min until it was closed. This viewer could prove a valuable alternative for users seeking better performance than Med3Web (although the loading speed for 3D will be slightly higher, the render will not be prone to freezing when altered), and are willing to overlook the time required for file uploads.

**Fig. 4:**
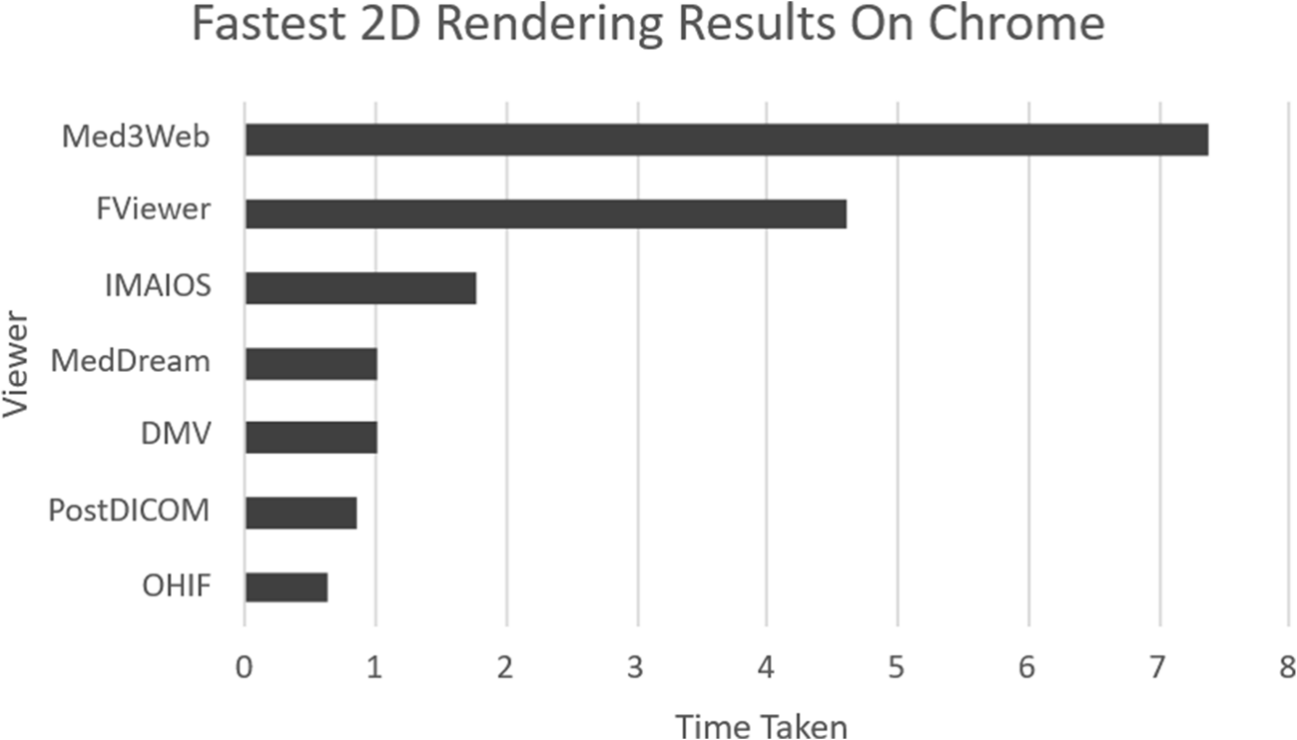
Chrome 2D rendering comparison chart

**Fig. 5:**
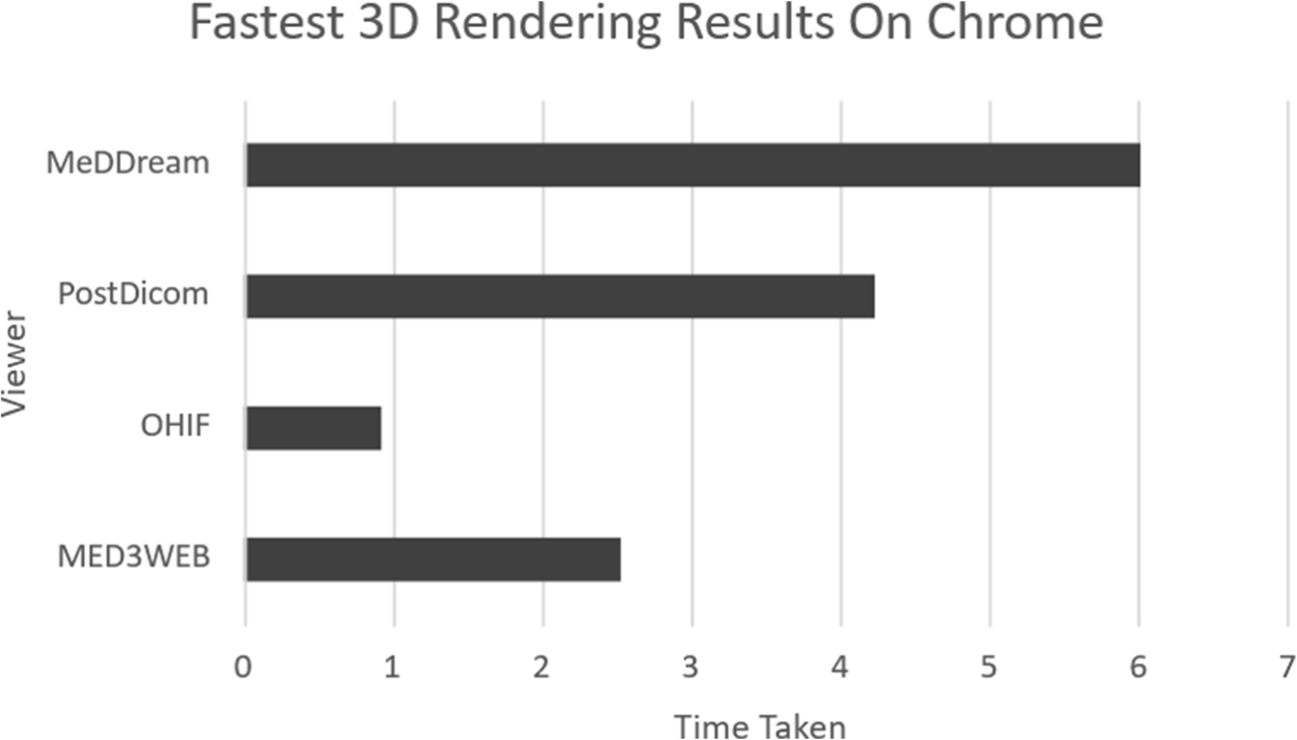
Chrome 3D rendering comparison chart

Notably, the evaluation revealed that MEDDream, PostDICOM, and OHIF demonstrated consistent improvements within the second 2D test, leveraging the advantages of browser caching. These viewers showcased better time intervals on the second 2D rendering test on most computers and browsers, enhancing efficiency over multiple sessions. On another note, the 3D rendering tests did not show a consistent improvement in time on the second test.

Hardware-wise, computer A, the computer with the best hardware, outperformed others in all browsers and viewers, with a single exception on PostDICOM, where computer C, arguably the weakest, had the best result. The improvement in performance from computer A to the others varied greatly, ranging from less than a second to minutes, depending on the viewer. The viewers most impacted by weaker hardware were IMAIOS and Med3Web. Conversely, the viewers less impacted by hardware were OHIF and PostDICOM.

As Chrome was the only browser that was run across all computers and did not fully fail any single viewer, it can be used for an overall comparison of all the viewers. This comparison can be seen in Fig. 4 for 2D rendering and Fig. 5 for 3D rendering.

From Figs. 4 and 5 it can be concluded that OHIF is the winner in terms of fast performance, however, it’s important to keep in mind the viewer’s setbacks in terms of 3D viewing, which are not present in the other two.

## Conclusion

The comprehensive evaluation of various DICOM viewers has provided valuable insights into their performance, usability, and features. Among the viewers tested, OHIF and PostDICOM emerged as the fastest among those with web apps available, with MEDDream lagging slightly behind in the 3D viewing area but still showcasing efficient 2D load times. OHIF, despite having its 3D rendering interface in development, demonstrated superior efficiency in the features it has implemented. Med3Web exhibited slower performance compared to OHIF on both 3D and 2D rendering, and compared to MEDDream and PostDICOM, it had slower 2D rendering speeds and lag when making alterations to the 3D render. However, faster rendering methods can be selected to increase speed and responsiveness. Additionally, unlike PostDICOM and MEDDream, it doesn’t require users to upload their files to the cloud before usage.

PostDICOM, on the other hand, comes with numerous features readily available and stands out as a versatile option with the second-best performance overall, and the best performance on weaker computers. However, it comes with a notable caveat — it is a paid service. MEDDream serves as a viable alternative that also offers a competitive number of features, including cloud storage. The inclusion of cloud access further extends their functionality but requires file uploads, which will be time-consuming, and depending on file size, and network upload speed, could add hours to the time needed for users to finally view the files. IMAIOS, DWV, and FViewer, on the other hand, demonstrated slower performance compared to OHIF, MEdDream, and PostDICOM. As they only offer 2D features, OHIF is the better option, as the other viewers need to improve in terms of speed and responsiveness to remain competitive.

Browser choice significantly influences the display speed, with Chrome and Firefox identified as the faster options. Furthermore, Windows machines generally outperformed their Mac counterparts. However, Safari performed similarly to Chrome when supported by the viewer. This insight is crucial for users seeking to optimize their viewing experience based on browser preferences.

In summary, the choice of a DICOM viewer depends on specific user requirements, preferences, and considerations. OHIF, with its speed, presents a compelling option for users prioritizing efficiency and performance. PostDICOM and MEDDream offer cloud access (via DICOMLibrary for MEDDream’s case) and are feature-complete viewers, making them the best choice for projects that require multiple sessions, provided the time needed for uploading is not a concern. For 3D rendering, Med3Web may be suitable for users willing to tolerate occasional stutters when interacting with their render, as a trade-off for avoiding long uploads to a cloud. When focusing on 2D viewing and fast results, OHIF emerges as the better option, offering features comparable to Weasis and PostDICOM, along with the fastest rendering available online for free.

## Data Availability

The data used in this study is free and publicly available [37]: https://medimodel.com/sample-dicom-files/class-3-malocclusion/

## Appendix A: Computer Specifications

- **Computer A:**
  1. Processor: AMD Ryzen 5 5600X 6-Core Processor
  2. Video Card: NVIDIA GeForce RTX 3080
  3. Operating System: Windows 11
  4. RAM: 16 GB
- **Computer B:**
  1. Processor: AMD Ryzen 5 5600 H with Radeon Graphics
  2. Video Card: NVIDIA GeForce RTX 3060 Laptop GPU
  3. Operating System: Windows 11
  4. RAM: 16 GB
- **Computer C:**
  1. Processor: Intel(R) Core(TM) i7-7700HQ CPU @ 2.80GHz
  2. Video Card: NVIDIA GeForce GTX 1060
  3. Video Card #2: Intel(R) HD Graphics 630
  4. Operating System: Windows 10
  5. RAM: 16 GB
- **Computer D:**
  1. Processor: 4,2 GHz Intel Core 7
  2. Video Card: Radeon Pro 580 8192 MB
  3. Operating System: macOS High Sierra version 10.13.6
  4. RAM: 16 GB 2400 MHz DDR4
- **Computer E:**
  1. Processor: 3 GHz 8-Core Intel Xeon E5
  2. Video Card: AMD firePro D700 6 GB
  3. Operating System: macOS Monterey version 12.6.3
  4. RAM: 16 GB 1866 MHz DDR3

## Appendix B: Time Breakdowns

### B.1 OHIF Time Breakdowns

**Table 10:** OHIF test 1 times

Browser	Computer	Breakdown (s)
Firefox	A	(00.25 + 00.93)
	B	(00.29 + 00.64)
	C	(00.49 + 01.50)
Edge	A	(00.56 + 00.80)
	B	(00.81 + 00.66)
	C	(01.58 + 01.03)
Chrome	A	(00.31 + 00.49)
	B	(00.63 + 00.61)
	C	(01.26 + 01.30)
	D	(01.18 + 01.20)
	E	(01.27 + 01.34)

**Table 11:** OHIF test 2 times

Browser	Computer	Breakdown (s)
Firefox	A	(00.20 + 00.64)
	B	(00.28 + 00.09)
	C	(00.64 + 00.34)
Edge	A	(00.62 + 00.85)
	B	(00.68 + 00.49)
	C	(01.52 + 00.63)
Chrome	A	(00.34 + 00.38)
	B	(00.49 + 00.15)
	C	(00.99 + 00.30)
	D	(01.07 + 0.24)
	E	(01.01 + 00.61)

### B.2 MED3WEB Time Breakdowns

**Table 12:** MED3WEB test 1 times

Browser	Computer	Breakdown (s)
Firefox	A	(07.56 + 01.76)
	B	(07.76 + 02.13)
	C	(24.13 + 04.13)
Edge	A	(90.54 + 61.65)
	B	(93.22 + 61.97)
	C	(179.85 + 129.29)
Chrome	A	(04.99 + 02.43)
	B	(05.50 + 02.72)
	C	(15.77 + 05.63)
	D	(10.48 + 05.08)
	E	(10.52 + 05.35)

**Table 13:** MED3WEB test 2 times

Browser	Computer	Breakdown (s)
Firefox	A	(09.04 + 01.49)
	B	(05.68 + 01.74)
	C	(19.46 + 04.36)
Edge	A	(90.75 + 61.22)
	B	(93.40 + 62.62)
	C	(180.96 + 121.66)
Chrome	A	(04.91 + 02.49)
	B	(12.29 + 02.70)
	C	(16.15 + 05.05)
	D	(10.57 + 05.02)
	E	(10.37 + 05.59)
